# MAPK14 over-expression is a transcriptomic feature of polycythemia vera and correlates with adverse clinical outcomes

**DOI:** 10.1186/s12967-021-02913-3

**Published:** 2021-05-31

**Authors:** Chao Guo, Ya-yue Gao, Qian-qian Ju, Min Wang, Chun-xia Zhang, Ming Gong, Zhen-ling Li

**Affiliations:** Department of Hematology, China-Japan Friendship Hospital, Yinghua East Street, Beijing, China

**Keywords:** Polycythemia vera, WGCNA, MAPK14, Expression

## Abstract

**Background:**

The transcriptomic signature has not been fully elucidated in PV, as well as mRNA markers for clinical variables (thrombosis, leukemic transformation, survival, etc.). We attempted to reveal and validate crucial co-expression modules and marker mRNAs correlating with polycythemia vera (PV) by weighted gene co-expression network analysis (WGCNA).

**Material and methods:**

The GSE57793/26014/61629 datasets were downloaded from Gene Expression Omnibus (GEO) database and integrated into one fused dataset. By R software and ‘WGCNA’ package, the PV-specific co-expression module was identified, the pathway enrichment profile of which was obtained by over-representation analysis (ORA). Protein–protein interaction (PPI) network and hub gene analysis identified MAPK14 as our target gene. Then the distribution of MAPK14 expression in different disease/mutation types, were depicted based on external independent datasets. Genome-scale correlation analysis revealed the association of MAPK14 and JAK/STAT family genes. Then gene set enrichment analysis (GSEA) was performed to detect the activated and suppressed pathways associating with MAPK14 expression. Moreover, GSE47018 dataset was utilized to compare clinical variables (thrombosis, leukemic transformation, survival, etc.) between MAPK14-high and MAPK14-low groups.

**Results:**

An integrated dataset including 177 samples (83 PV, 35 ET, 17 PMF and 42 normal donors) were inputted into WGCNA. The ‘tan’ module was identified as the PV-specific module (R^2^ = 0.56, p = 8e−16), the genes of which were dominantly enriched in pro-inflammatory pathways (Toll-like receptor (TLR)/TNF signaling, etc.). MAPK14 is identified as the top hub gene in PV-related PPI network with the highest betweenness. External datasets validated that the MAPK14 expression was significantly higher in PV than that of essential thrombocytosis (ET)/primary myelofibrosis (PMF) patients and normal donors. JAK2 homozygous mutation carriers have higher level of MAPK14 than that of other mutation types. The expression of JAK/STAT family genes significantly correlated with MAPK14, which also contributed to the activation of oxidated phosphorylation, interferon-alpha (IFNα) response and PI3K-Akt-mTOR signaling, etc. Moreover, MAPK14-high group have more adverse clinical outcomes (splenectomy, thrombosis, disease aggressiveness) and inferior survival than MAPK14-low group.

**Conclusion:**

MAPK14 over-expression was identified as a transcriptomic feature of PV, which was also related to inferior clinical outcomes. The results provided novel insights for biomarkers and therapeutic targets for PV.

**Supplementary Information:**

The online version contains supplementary material available at 10.1186/s12967-021-02913-3.

## Background

Classic Philadelphia negative myeloproliferative neoplasms (MPN) is constituted by polycythemia vera (PV), essential thrombocythemia (ET) and primary myelofibrosis (PMF) [1]. The incidence of PV reported as 1.03 per 100,000 per year, respectively [2, 3]. PV is characterized by aberrantly over-erythrocytosis, and almost all patients harbor JAK2V617F mutation. IFNα and hydroxyurea are the traditional treatment for MPN patients with excessive blood cells. The COMFORT I and II trials established the role of JAK2 inhibitor, ruxolitinib, in advanced MPNs. But for many patients, the failure of JAK2 inhibitor is still a major challenge. Therefore, numerous novel target drugs were developed and ongoing clinical trials [4], such as PI3K inhibitors, heat shock protein inhibitors, HDAC inhibitors, etc. However, biomarkers associating with clinical outcomes and efficacy of target therapy are still insufficient, which can evaluate the activation of JAK2 and predict the sensitivity to drugs.

Several studies have been conducted for the transcriptomic features of PV. After the identification of differentially expressed genes (DEG) between PV patients and normal donors, ORA resulted in the enriched pathways correlating with the disease or mutation types, which promoted the finding of novel biomarkers [5–10]. WGCNA was an updated method of transcriptomic analysis, which identified the co-expression modules based on the established scale-free network and analyzed the correlation of the clinical/genetic traits and module eigengenes (ME) (the first principal component of gene co-expression modules) [11, 12]. Thus, we can obtain the co-expression modules specifically correlated with PV, which then were inputted into ORA and PPI analysis. By the results of our analysis, MAPK14, an essential component of the MAP kinase signal transduction pathway, was identified as the top hub gene of PV-specific module and involved in several pro-inflammatory pathways. By WGCNA, this study uncovered and validated a promising disease biomarker and therapeutic target for PV.

## Methods

### Data source

From GEO online database (https://www.ncbi.nlm.nih.gov/gds/), the GSE61629/GSE26049/GSE57793 datasets were obtained. All the 3 datasets were performed on the same platform (GPL570, Affymetrix Human Genome U133 Plus 2.0 Array) and originated from the same cell origin (peripheral blood cells). In order to obtain as many samples as possible to improve statistic power of WGCNA [13], we integrated them into one large, fused expression matrix, after removing batch effects using ‘combat’ function of R software (version 4.0.2). The fused matrix included transcriptomic information from 177 patients (35 ET, 83 PV, 17 PMV and 42 normal donors).

GSE103237 dataset included transcriptomic data from bone marrow CD34+ cells of 65 patients (24 ET, 26 PV and 15 normal donors). GSE54644 dataset included transcriptomic data from peripheral neutrophiles of 93 patients (47 ET, 28 PV, 18 PMF and 11 normal donors). GSE103237/GSE54644 were utilized to validate the expression difference of MAPK14 between different disease/mutation types. To investigate the association of MAPK14 expression and clinical variables, clinical information and MAPK14 expression data were extracted from GSE47018, which included 20 PV patients and 7 normal donors. The clinical characteristics and application in this study were listed in Table 1.

**Table 1:** The summary of GEO datasets used in the present study

GEO accession	Number of samples	ET	PV	PMF	Normal donors	Application in this study
GSE61629 [10]	21	0	0	0	21	WGCNA to reveal hub genes correlating with PV
GSE26049 [7]	90	19	41	9	21	WGCNA to reveal hub genes correlating with PV
GSE57793 [8]	66	16	42	8	0	WGCNA to reveal hub genes correlating with PV
GSE103237 [5]	65	24	26	0	15	Validation for the correlation of hub gene expression with MPN subtypes and mutation types
GSE54644 [6]	104	47	28	18	11	Validation for the correlation of hub gene expression with MPN subtypes and mutation types
GSE47018 [9]	27	0	20	0	7	Validation for the correlation of hub gene expression with clinical variables in PV

The expression value was commonly log2-transformed before manipulation, which made the standard deviation (SD) independent of data magnitude and fit to the normal distribution better [2]. Therefore, the log2-transformed data was utilized in correlation analysis, such as WGCNA and GSEA. While to depict the absolute difference of MAPK14 expression between disease/mutation subgroups, the non-transformed value was analyzed.

### WGCNA

The genome-scale transcriptomic data was implemented to construct the co-expression network, using ‘WGCNA’ package of R software (version 4.0.2) [11]. To reveal inter-individual heterogeneity and detect outliers, hierarchical clustering method was used based on average linkage. The soft threshold power was defined as minimal beta value with scale free R^2^ > 0.85. The inter-gene correlation coefficients by Pearson’s method, was computed to establish the matrix of gene adjacency and topological overlap matrix (TOM). The hierarchical clustering according to average linkage, was used to divide the whole transcriptome into co-expression modules. The modules with less than 25% dissimilarity were merged together. And the minimal size of gene co-expression module was set as 30 genes. MEs were defined as the principal component of individual co-expression modules. Module membership (MM) was defined as the Pearson’s correlation coefficient between the corresponding ME and individual gene expression value. Gene significance (GS) was defined as the Pearson’s correlation coefficients between the trait (disease type of MPN) and individual gene expression value. PV-specific module was defined as the module, which had the highest correlation coefficient with PV, and was statically irrelevant or negatively correlated with other disease types (ET/PMF/normal donors). Within the PV-specific module, the genes with MM ≥ 0.8, weighted q value < 0.01, and GS ≥ 0.2, was identified as hub genes.

### ORA and PPI network analysis of the PV-specific module

To illustrate the impact on cell signaling by the PV-specific module, Database for Annotation, Visualization and Integrated Discovery (DAVID) (https://david.ncifcrf.gov/) was implemented for ORA based on the Kyoto Encyclopedia of Genes and Genomes (KEGG) database [14]. The local FDR adjusted p value (q value) < 0.05 is set as cut-off value for significant enriched pathways.

To establish PPI network based on the previous evidence and experiments, Search Tool for the Retrieval of Interacting Genes (STRING) database [15] (https://string-db.org/) was used for hub genes in the ‘tan’ module. The hub genes were inputted into STRING database, PPI pairs of which were extracted with interaction score > 0.4. The betweenness of nodes were computed and ranked by cytoscape software (version 3.7.2) and cytohubba plugin.

### Validation of MAPK14 expression difference between disease/mutation types

To validate the association of MAPK14 expression with disease/mutation types, non-transformed MAPK14 expression value was obtained from 3 independent datasets (the fused GSE61629/GSE26049/GSE57793 dataset, GSE103237 and GSE54644), respectively. The distribution of MAPK14 expression was investigated for MPN subtypes (PV/ET/PMF) and normal donors. The patients with distinct mutation types (JAK2/CALR, etc.) were compared as well.

### Whole transcriptome correlation analysis of MAPK14

Based on transcriptomic data of the abovementioned fused dataset, the correlation analysis was performed to uncover genes significantly correlated with MAPK14 by Pearson’s method. The MAPK14 related genes were defined as p value < 0.05. Then, GSEA was performed to evaluate the activation/suppression of MAPK14 related genes on cell signaling pathways, based on MSigDB database [24–26] (http://software.broadinstitute.org/gsea/msigdb). The significantly activated/suppressed signaling pathways were defined as |normalized enrichment score (NES)|> 1 and q value < 0.05.

### The association of MAPK14 and clinical variables

The expression value of MAPK14 was obtained from GSE47018 dataset. PV patients were dichotomized into MAPK14-low and MAPK14-high groups by the median value of MAPK14 expression. Clinical variables including age, JAK2 mutation burden, initiation of drug therapy, hemoglobin, white blood cells count, platelet, splenectomy, thrombosis, acute myeloid leukemia (AML) transformation, disease aggressiveness and survival. According to the description original article [9], the study corresponding to GSE47018, was initiated in 2003 and terminated in 2012. The end of 2012 was the time point, at which the survival status of patients was confirmed. In addition, the unsupervised hierarchical clustering based on transcriptomic data of GSE47018, segregated patients into 2 groups with different disease behaviors (disease duration, JAK2V617F mutation burden, thrombosis, leukemic transformation, survival, etc.) [9], which were defined as aggressive and indolent groups, respectively. The definition of disease aggressiveness was consistent with description of the original study, the detail on which was depicted in the supplementary data of GSE47018.

### Statistical analysis

If the data deviated from normal distribution (log2 transformed data), unpaired t test was used to compare the continuous variables of subgroups. Otherwise, the Mann–Whitney test was used for non-transformed data. The ordinary one-way ANOVA test was utilized to compare variables of more than 2 groups. The bilateral Fisher exact test was employed to compare categorical variables of subgroups.

## Results

### Results of WGCNA

**Fig. 1:**
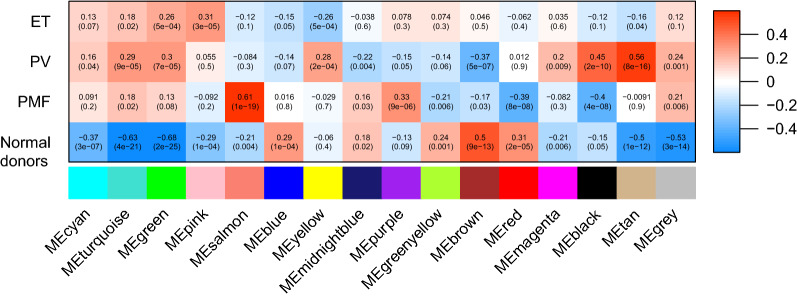
The relationship of gene co-expression clusters and disease subtypes. All modules (colors) are displayed on the X axis, while all disease subtypes are displayed on the Y axis. Each box contains corresponding Pearson’s coefficients (by gradient of color, red = 1, blue =  − 1) and p value

**Fig. 2:**
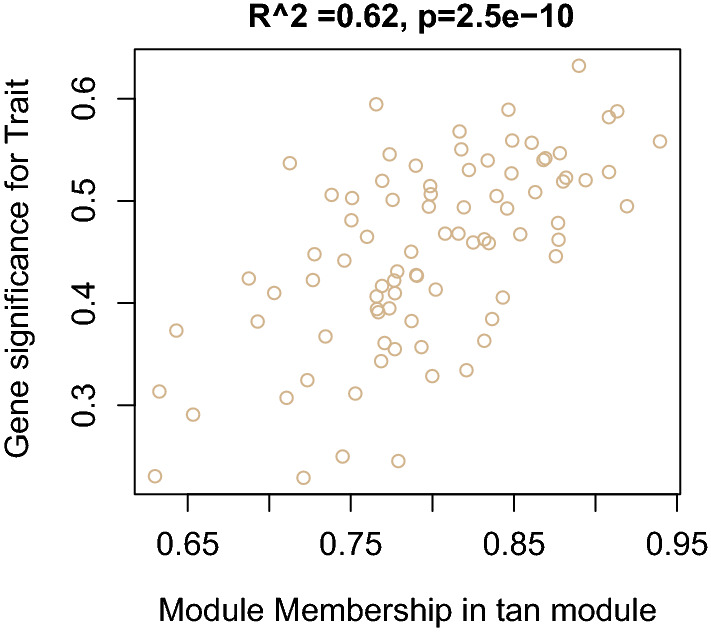
The correlation of MM and GS for the ‘tan’ module, in which individual dots stand for one specific gene

After all samples were hierarchically clustered, no obvious inter-individual heterogeneity was detected, since no outliers were found among the 3 datasets (Additional file 1: Figure S1). 16 was set as the soft threshold power, according to the scale free R^2^ distribution (Additional file 2: Figure S2). The whole gene was divided into 32 gene modules according to TOM-based dissimilarity (Additional file 3: Figure S3). The heatmap of topological overlap to reveal correlation between 400 randomly selected genes from different modules was shown in Additional file 4: Figure S4, which indicated topological overlap degree of individual modules. Moreover, ME adjacency heatmap illustrated the relationship of modules (Additional file 5: Figure S5). Finally, the relationship of modules and disease types (PV/ET/PMF/normal donors) was demonstrated in Fig. 1. The ‘tan’ module was identified as the PV-specific module (R^2^ = 0.56, p = 8e−16). Notably, the ‘tan’ module was irrelevant toPMF (p = 0.9), and negatively correlated with ET (R^2^ = − 0.16, p = 0.04)/ normal donors (R^2^ = − 0.6, p = 1e−12). 85 genes were included in the ‘tan’ co-expression module in total, 35 of which were identified as hub genes (listed in Additional file 8: Table 1). The correlation of MM and GS for individual genes of the ‘tan’ module was demonstrated in Fig. 2, with Pearson’s coefficient = 0.62 and p value = 2.5e−10, suggesting significant association between the ‘tan’ eigengene and disease types.

### ORA and PPI analysis for hub genes of the PV-specific module

**Fig. 3:**
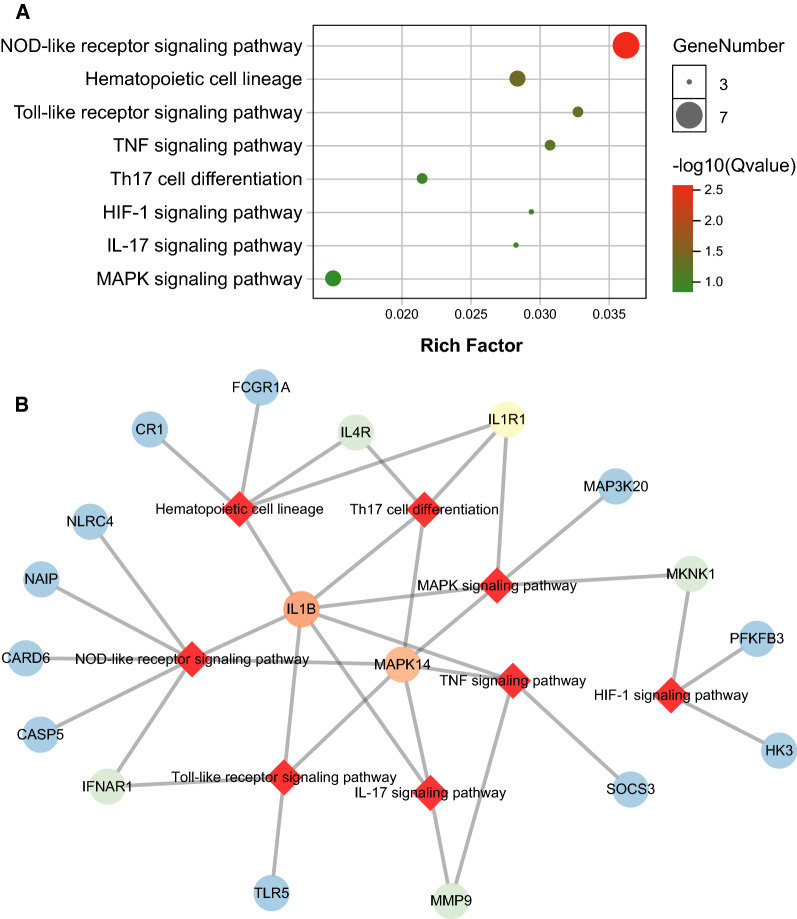
The results of ORA for PV-specific gene module. **A** The dotplot of enriched pathways. The size of dots represented the count of genes involved in the pathway. While the color of dots correlated with the -log10(q value). **B** The network of interaction between signaling pathways and hub genes within the PV-specific module, in which the circles represented hub genes and red rhombuses stand for enriched pathways. The color gradient of circles correlated with the connectivity degrees of individual genes (red for high degrees, blue for low degrees)

**Fig. 4:**
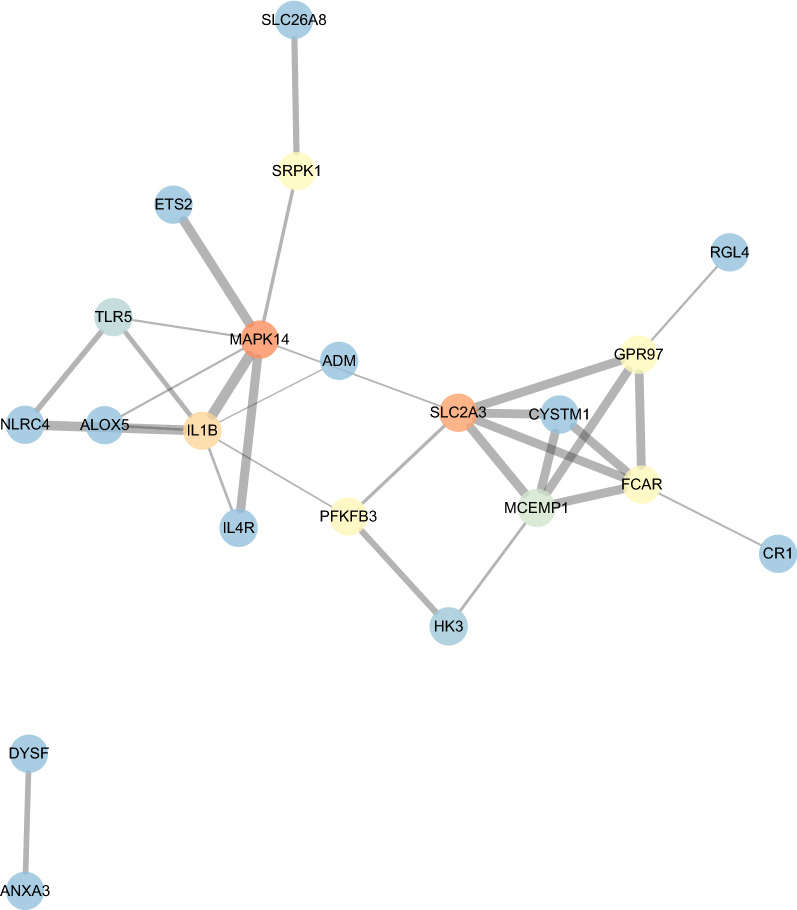
The PPI network for the PV-specific module. The color gradient of node indicated the betweenness (red for high betweenness, blue for low betweenness)

Based on KEGG database, the hub genes dominantly enriched in NOD-like receptor signaling, TLR signaling, TNF signaling, HIF-1 signaling, etc. (Fig. 3A). The network of genes and pathways relationship was demonstrated in Fig. 3B. After the hub genes were inputted into STRING database, PPI network was shown in Fig. 4. Top 5 genes with highest betweenness among the PPI network were MAPK14, SLC2A3, IL1B, PFKFB3, PCAR.

The ‘chooseTopHubInEachModule’ function of ‘WGCNA’ package, was used to identify the top hub genes in each module, which selected MAPK14 in the ‘tan’ module with the highest MM value. In combination with the result from ORA and PPI analysis, MAPK14 were chosen as the target gene in the following analysis.

### Validation for expression difference of MAPK14

**Fig. 5:**
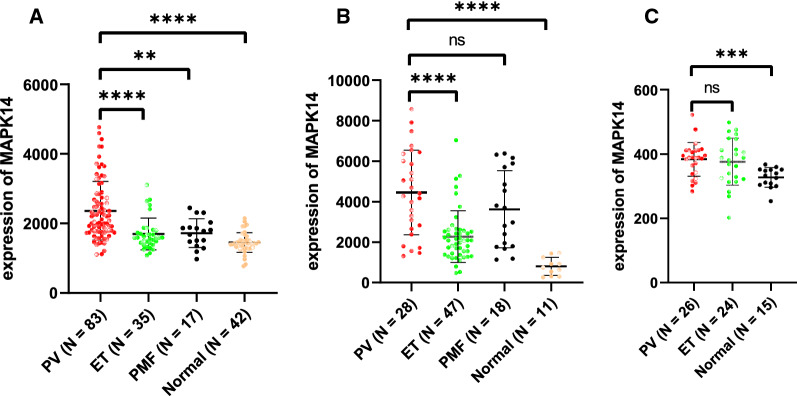
The normalized expression value of MAPK14 in different disease types of MPN and normal donors for the fused dataset (**A**), GSE54644 (**B**) and GSE103237 (**C**). *, p < 0.05; **, p < 0.01; ***, p < 0.001; ****, p < 0.0001

The Fig. 5A/B/C were dot plots for the non-transformed expression value of MAPK14 in different disease types, indicating MAPK14 expression level was significantly higher in PV patients than that of other MPN subtypes and normal donors (p < 0.05). Moreover, this result validated that the intrinsic MAPK14 over-expression was PV specific, no matter in peripheral neutrophils (GSE54644, Fig. 5B) or in bone marrow CD34+ cells (GSE103237, Fig. 5C).

**Fig. 6:**
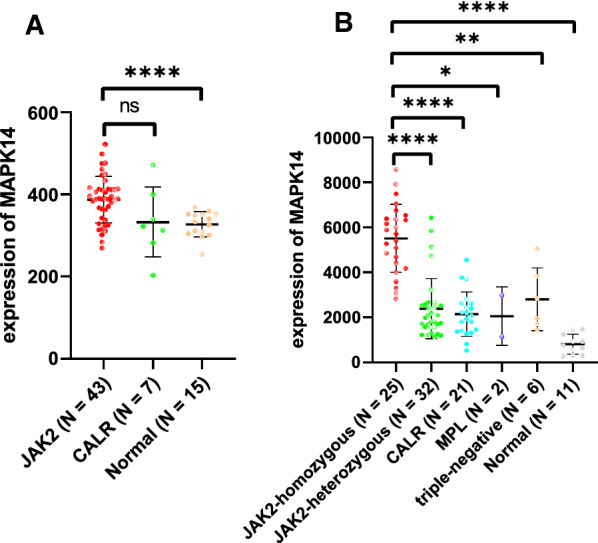
The normalized expression value of MAPK14 in different mutation types of MPN and normal donors for GSE103237 (**A**) and GSE54644 (**B**). *, p < 0.05; **, p < 0.01; ***, p < 0.001; ****, p < 0.0001

Since the mutation types of 7 patients were missing, 97 patients were taken into analysis in GSE54644. The Fig. 6A (GSE54644)/6B (GSE103237) were dot plots depicting the non-transformed expression value of MAPK14 for different mutation types. In Fig. 6A, significant higher MAPK14 expression level was found in JAK2 mutation carriers than that of normal donors (p < 0.05) instead of CALR mutation carriers. Notably, JAK2-homozygous mutation carriers had significantly higher expression level of MAPK14 than that of all other mutation types MPN patients and normal donors (p < 0.05, Fig. 6B). While no significant difference was uncovered between JAK2 heterozygous and CALR mutation (p = 0.9068), MPL mutation (p = 0.8021), and tri-negative (p = 0.4216) groups. Still, the JAK2 heterozygous mutation groups had significant higher MAPK14 expression than that of normal donors (p < 0.0001). The result indicated that increased MAPK14 expression was related to copy number of JAK2 mutation.

Additionally, to validate the diagnostic utility of MAPK14 expression for PV, ROC (receiver operating characteristic) curve was used to illustrate diagnostic utility (AUC) to distinguish PV with other MPNs/normal donors (Additional file 7: Figure S7).

### The association of JAK/STAT family genes with MAPK14

**Fig. 7:**
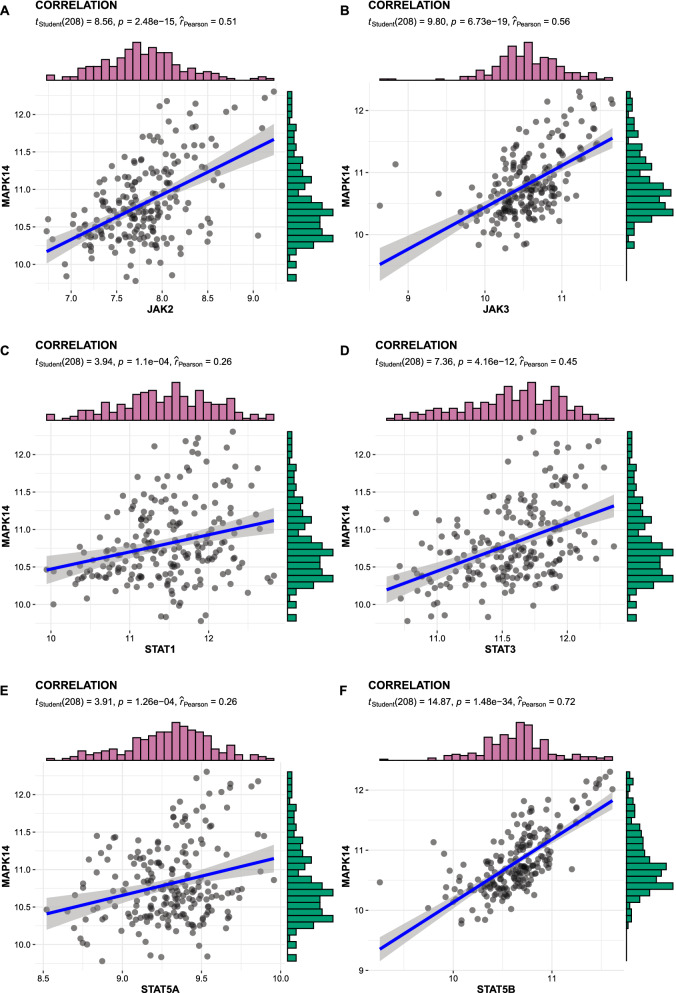
The transcriptomic correlation of MAPK14 and JAK/STAT family genes by the fused dataset

1199 genes were found to be related with MAPK14 on the level of expression significantly (p < 0.05). In JAK family genes, JAK2 and JAK3 expression were positively correlated with MAPK14 (R = 0.51 and 0.56, respectively; p = 2.48e−15 and 6.73e−19, respectively). In STAT family genes, STAT1, STAT3, STAT5A and STAT5B were positively associated with MAPK14 (Fig. 7), while STAT4 was negatively correlated with MAPK14 (R = − 0.53, p = 4.16e−16).

### The results of GSEA for MAPK14

**Fig. 8:**
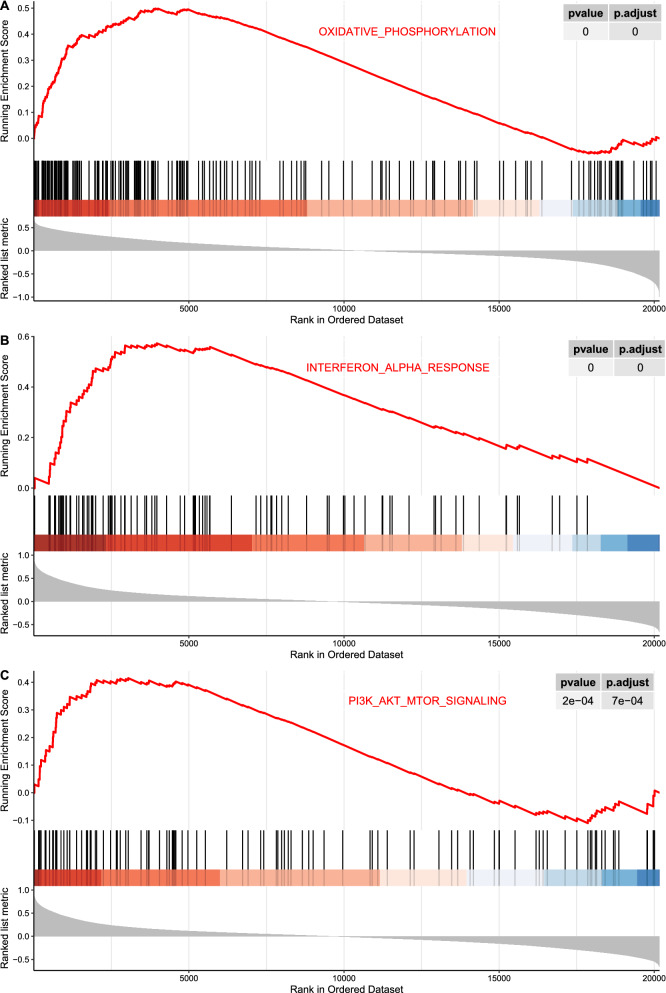
The curves of running enrichment score for MAPK14-related pathways

GSEA revealed the activated and suppressed signaling pathways associating with MAPK14 expression (Additional file 6: Figure S6). The activated pathways included oxidative phosphorylation, IFNα response, PI3K-Akt-mTOR signaling (Fig. 8). The suppressed signaling pathways included MYC targets, E2F targets, etc.

### Comparison of clinical variables between MAPK14-high and MAPK14-low groups

The results of analysis on GSE47018 were shown in Table 2. The proportion of patients who had initiated drug therapy, was higher in MAPK14-high group than that of MAPK14-low group (80% vs 10%, p = 0.005). According to the description original article [9], all patients of this cohort had received treatment (hydroxyurea, phlebotomy etc.), which lead to lower hemoglobin level.No significant difference was detected for hemoglobin, WBC or platelet. MAPK14 over-expression was associated with symptomatic PV, MAPK14-high group had significantly more patients with splenectomy (50% vs 0, p = 0.033) and thrombosis (50% vs 0, p = 0.033). Poorer prognosis and more aggressive disease were revealed in MAPK-high group, with significant lower survival ratio (40% vs 90%, p = 0.05) and more disease aggressiveness (70% vs 0, p = 0.003). To illustrate the predictive value of MAPK14, other key genes involving JAK2 signaling (JAK2/EPOR/STAT5A/STAT5B) were taken into consideration, the results of comparison of corresponding over-expression and under-expression groups were shown in Table 2 as well.

**Table 2:** The comparison of clinical variables between MAPK14-high and MAPK14-low groups, which were also performed for over-expression/under-expression subgroups of JAK2, EPOR, STAT5A and STAT5B. The p value ≤ 0.05 was in boldface

	MAPK14 low (n = 10)	MAPK14 high (n = 10)	p value (MAPK14-high vs low subgroups)	p value (JAK2-high vs low subgroups)	p value (EPOR-high vs low subgroups)	p value (STAT5A-high vs low subgroups)	p value (STAT5B-high vs low subgroups)
Age (year)	65.5	63.1	0.579	0.481	0.281	0.393	0.796
JAK2 mutation burden (%)	78.9	90.5	0.19	0.739	0.393	0.52	0.739
Initiation of drug therapy (%)	10	80	**0.005**	0.37	0.37	1	1
Hemoglobulin (g/dL)	13.1	11.62	0.063	0.684	0.796	0.853	0.631
Leukocyte count	16706	35999	0.529	0.631	0.684	0.19	1
Platelet count	741.3	648.5	0.684	0.971	0.19	0.853	0.971
Splenectormy (%)	0	50	**0.033**	1	1	1	1
Thrombosis (%)	0	50	**0.033**	1	1	1	1
AML transformation (%)	10	40	0.303	1	1	1	1
Disease aggressiveness (%)	0	70	**0.003**	1	1	1	1
Survival (%)	90	40	**0.05**	1	0.35	1	1

## Discussion

The abnormality of blood cells and complications of PV patients can be controlled by hydroxyurea, interferons and JAK2 inhibitors in clinical practice. While the failure of primary treatments in advanced stage is still challenging by far. Exploring the expression signature by WGCNA provided us a novel way to understand the expression changes correlating with the disease or mutations, which may help us to reveal novel predictive biomarkers and therapeutic targets.

The gene co-expression network analysis identified the ‘tan’ module as PV-specific gene cluster (Fig. 1). Genes of the ‘tan’ module was predominantly enriched in TLR signaling, TNF signaling, Th17 cell differentiation, IL17 signaling, NOD-like receptor signaling, hematology cell linage, HIF- signaling and MAPK signaling (Fig. 3). Inflammation is an important pathogenetic driver in MPN. Over-expressed pro-inflammatory cytokines including TNFα [16, 17] and IL1β [18, 19], were revealed in PV and correlated with JAK2V617 mutation burden. Hew Yeng Lai et al. found that the negative feedback regulation of TLR signaling, resulting in over-production of TNFα by monocytes in MPN patients [20]. The increased TNFα and activated downstream signaling promoted clone expansion of neoplastic cells of MPN in return [21]. Th17 cells, secreting IL17, played a crucial role in tumor immune surveillance [22], and was raised after JAK inhibitor treatment in MPN patient, especially in clinically responded patients [23], suggesting genes involving Th17 cell differentiation and IL17 signaling in the ‘tan’ module played a role in immune microenvironment of PV. Although it’s not studied in MPN, NOD-like receptor proteins were reported to be involved in inflammatory pyroptosis and immune disorder in bone marrow microenvironment, which were the crucial drivers in MDS pathogenesis [24]. Among the hub genes of PV-specific module, MAPK14 and IL1β were involved in all above-mentioned pathways (Fig. 3B), suggesting they played a key role in pro-inflammatory status in MPN patients. HIF1 were found to be the master regulator in the circumstance of low oxygen level, and inhibitor of HIF-1 by shRNA or echinomycin suppressed cell growth and induced apoptosis in JAK2 mutated cells instead of JAK2 wild-type cells [25]. These findings supported that genes of the ‘tan’ module involved in the pivotal pathways in PV.

MAPK14, as one of p38 proteins, participated in both canonical and non-canonical (TLR signaling, TNF signaling, etc.) JAK2 downstream signaling [26]. In combination with ORA and PPI analysis, MAPK14 was identified as the top hub gene in the ‘tan’ module and chosen as the target gene in the study. MAPK14, encoding Mitogen-activated protein kinase 14, played a role in erythropoiesis by stabilizing the EPO mRNA in the mice model [27]. Based on the results using external independent datasets, the expression level of MAPK14 was higher in PV, than that of ET/PMF/normal donors. This conclusion was consistent in both peripheral neutrophils (Fig. 5A/B) and bone marrow CD34+ cells (Fig. 5C), indicating it was an intrinsic transcriptomic feature across the hematopoietic cell lineage of PV patients. The results of ROC analysis indicated that MAPK14 was a good diagnosis indicator for PV patients, based on the AUC value.

PV patients with gain of mutated JAK2 copies (trisomy 9, or other chromosomal 9 aberrations) had upregulated MAPK14 expression [28]. Since the JAK2 mutation rate was substantially higher in PV (96%) than that of ET (55%) or PMF (65%) according to the previous research [29–32]. The association of PV with MAPK14 expression may virtually be attributed to the higher frequency of JAK2 mutation in MPN types. Therefore, to investigate the relationship of mutation types and MAPK14 expression, the distribution of expression value was depicted in Fig. 6. For GSE103237 (Fig. 6A), JAK2 mutated patients had significantly higher MAPK14 expression than normal donors, while no significant difference was found between JAK2 mutated and CALR mutated patients. Based on GSE54644, JAK2 homozygous mutated patients had higher expression of MAPK14 than all other mutation types of MPN and normal donors (Fig. 6B), but no significant difference were uncovered between JAK2 heterozygous mutation and other mutation types, which suggested number of mutated JAK2 copies was correlated with MAPK14 expression. To further investigate the quantitative correlation of MAPK14 and JAK2/STAT genes, global expression profile correlating with MAPK14 expression, uncovered that JAK (JAK2/JAK3) and STAT (STAT1/STAT3/STAT5A/STATA5B) family genes were positively correlated with MAPK14 in MPN patients (Fig. 7). To specific disease types of MPN, MAPK14 significantly positively correlated with JAK2 expression in PV and ET patients respectively (PV: p = 2.35e−8, Pearson’s coefficient = 0.57; ET: p = 0.037, Pearson’s coefficient = 0.35). Therefore, MAPK14 expression is not only correlated to allelic status of JAK2V617F mutation, but also the mRNA level of JAK/STAT genes. MAPK14 was a promising parameter for activated downstream signaling of mutated JAK2.

The following GSEA indicated that MAPK14 was associated with activation of oxidative phosphorylation/IFN-α response/PI3K-Akt-mTOR signaling, etc. Metabolic aberrations had been investigated in MPN. Tata Nageswara Rao et al. founded that mutated JAK2 resulted increased glycolysis and oxidative phosphorylation in MPN cells [33]. PFKFB3 was the key regulator of glycolysis, inhibitor of which reversed the altered metabolic status including excessive oxidative phosphorylation and glycolysis in vitro [33], which was significantly correlated with MAPK14 (Pearson’s coefficient = 0.80, p = 1.14e−4) and a hub gene of the ‘tan’ module (Fig. 3 and 4). So, the association of metabolic alterations with MAPK14 expression, may contribute to the inferior clinical outcomes of MAPK14-high patients, and provided novel targets. Interferon-alpha (IFNα) is an effective treatment for MPN patients, including PV. IFNα promoted the shift towards CD41(ITGA2B) high subset of hematopoietic stem cells in JAK2V617F clones [34], exerting therapeutic effects. Notably, CD41 and MAPK14 were significantly co-expressed (Pearson’s coefficient = 0.25, p = 2.85e−4), and MAPK14 expression positively correlated with IFNα response pathway (Fig. 8). MAPK14 can be a possible marker to predict response of IFNα therapy for PV patients. PI3K-Akt-mTOR signaling correlated with MAPK14 expression positively (Fig. 8), pharmacological inhibitor of which had been preliminarily investigated and showed some effects on MPN in several studies [35–37]. Notably, MAPK14, encoding p38α, lied in the ERK/MEK signaling, inhibition of which had been found to be synergistic with ruxolitinib in JAKV617F mice [38]. Since STAT/MAPK/PI3K-Akt-mTOR pathways were main downstream signaling of JAK2 activation [26], additive PI3K-Akt-mTOR and/or MAPK/MEK may potentially increase JAK2 inhibitor efficacy in MAPK14-high patients.

Clinical variables were compared in MAPK14-high and MAPK14-low groups (Table 2). The JAK2 mutation burden was insignificantly higher in MAPK14-high group compared to MAPK14-low group (90.5% vs 78.9%, p = 0.19), which may be due to insufficient samples. Moreover, in comparison with MAPK14-low group, MAPK14-high group had significantly inferior clinical outcomes (Table 2). Mutated JAK2 protein was reported to bind to EPOR and promoted activation of STAT5 in PV patients [39]. So, the patients from GSE47018 were dichotomized into over-expression and under-expression subgroups of JAK2/EPOR/STAT5A/STAT5B, respectively. Surprisingly, the clinical parameters showed no significant difference (Table 2). These results suggested that MAPK14 expression was a promising marker for PV in clinical practice, instead of other JAK2 signaling genes.

## Conclusion

MAPK14 was demonstrated to lie in the central position of PPI network and PV-related pro-inflammatory pathways. The MAPK14 expression level was not only correlated to PV, but also correlated to the allelic status/mRNA quantity of JAK2. MAPK14-high PV patients had more symptoms and inferior clinical outcomes. This study identified MAPK14 as a promising disease marker and provided insight into therapeutic targets.

## Supplementary Information

**Additional file 1: Figure S1.** The results of sample clustering by average linkage hierarchical clustering method.

**Additional file 2: Figure S2.** The value of scale independence (left) and mean connectivity (right) to identify the soft threshold in the following network analysis.

**Additional file 3: Figure S3.** The cluster dendrogram (upper) and gene co-expressed modules (lower), in which the height of branches represented the distance of Euclidean.

**Additional file 4: Figure S4.** The topological overlap heatmap for 400 randomly selected genes.

**Additional file 5: Figure S5.** The module eigengene adjacency heatmap (lower), which indicated the relationship between distinct co-expression modules. The results of module clustering were shown in the upper part.

**Additional file 6: Figure S6.** The dotplot of GSEA results associating with MAPK14 expression. The size of dots represented the count of genes involved in the corresponding pathways. While the color of dots correlated with the -log10(adjusted p value).

**Additional file 7: Figure S7.** ROC curve of MAPK14 expression on diagnosis of PV based on the fused GSE61629/GSE26049/GSE57793 dataset (A), GSE54644 (B), and GSE103237 (C).

**Additional file 8: Table S1.** The list of hub genes in the ‘tan’ module.

## Data Availability

The data that support the findings of this study are available from GEO database (https://www.ncbi.nlm.nih.gov/gds/), which are all publicly available.
