# Supplementary figures and images for: MAPK14 over-expression is a transcriptomic feature of polycythemia vera and correlates with adverse clinical outcomes
Source: J Transl Med. 2021 May 31;19:233. doi: 10.1186/s12967-021-02913-3 (PMC8166116; doi:10.1186/s12967-021-02913-3)

Sample clustering to detect outliers

Height

20 40 60 80 100

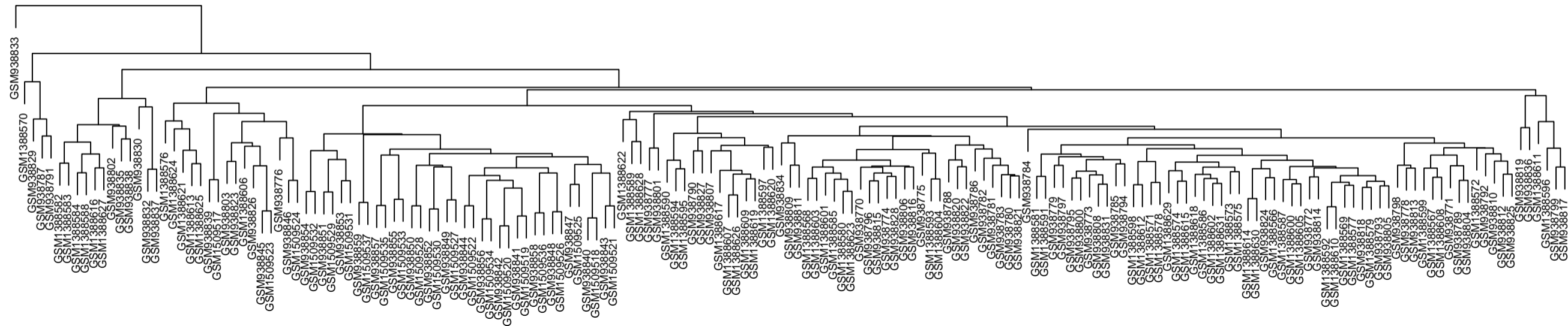

Supplement: Supplementary file 1 — Additional file 1: Figure S1. The results of sample clustering by average linkage hierarchical clustering method. [file 12967_2021_2913_MOESM1_ESM.pdf]

## Scale independence

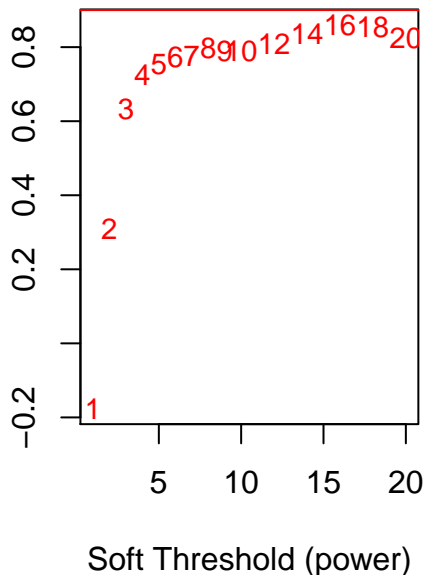

## Mean connectivity

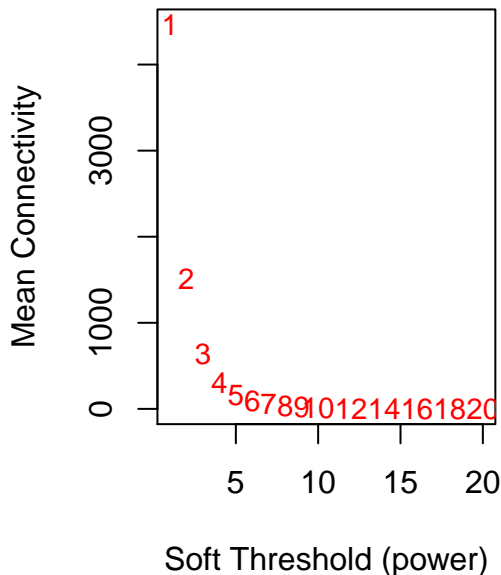

Supplement: Supplementary file 2 — Additional file 2: Figure S2. The value of scale independence (left) and mean connectivity (right) to identify the soft threshold in the following network analysis. [file 12967_2021_2913_MOESM2_ESM.pdf]

# Cluster Dendrogram

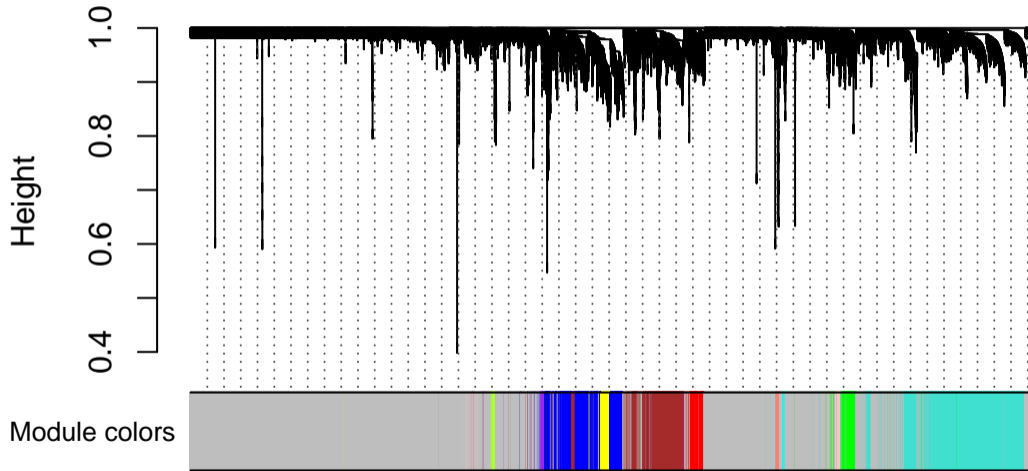

Supplement: Supplementary file 3 — Additional file 3: Figure S3. The cluster dendrogram (upper) and gene co-expressed modules (lower), in which the height of branches represented the distance of Euclidean. [file 12967_2021_2913_MOESM3_ESM.pdf]

Network heatmap plot, selected genes

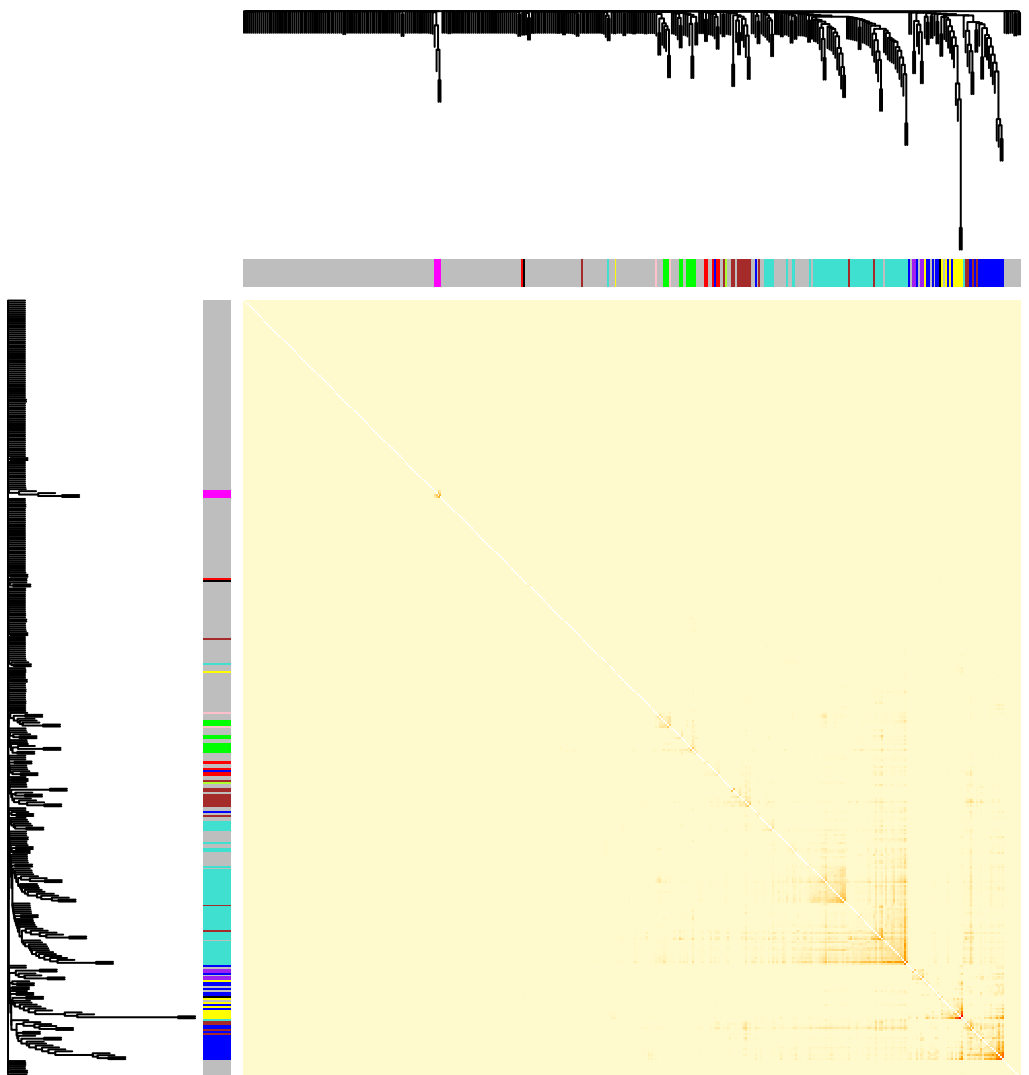

Supplement: Supplementary file 4 — Additional file 4: Figure S4. The topological overlap heatmap for 400 randomly selected genes. [file 12967_2021_2913_MOESM4_ESM.pdf]

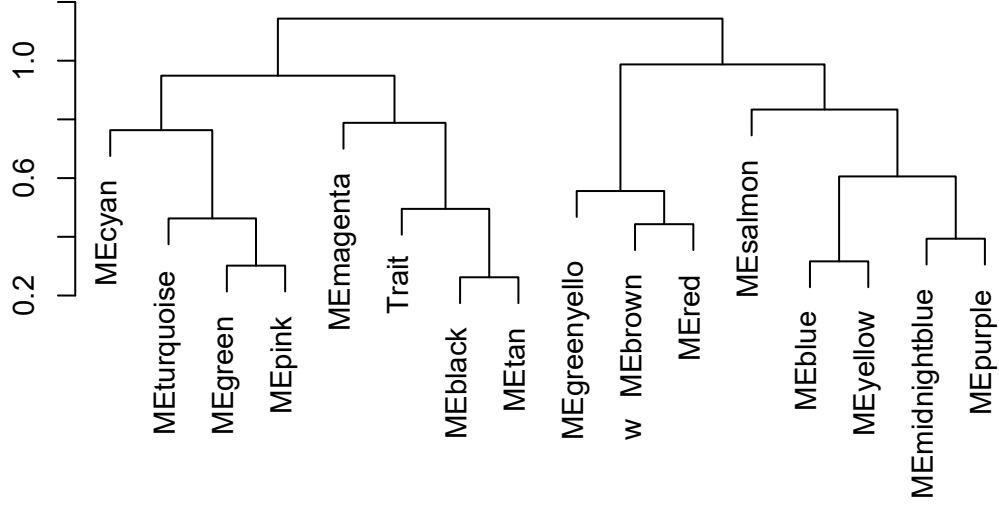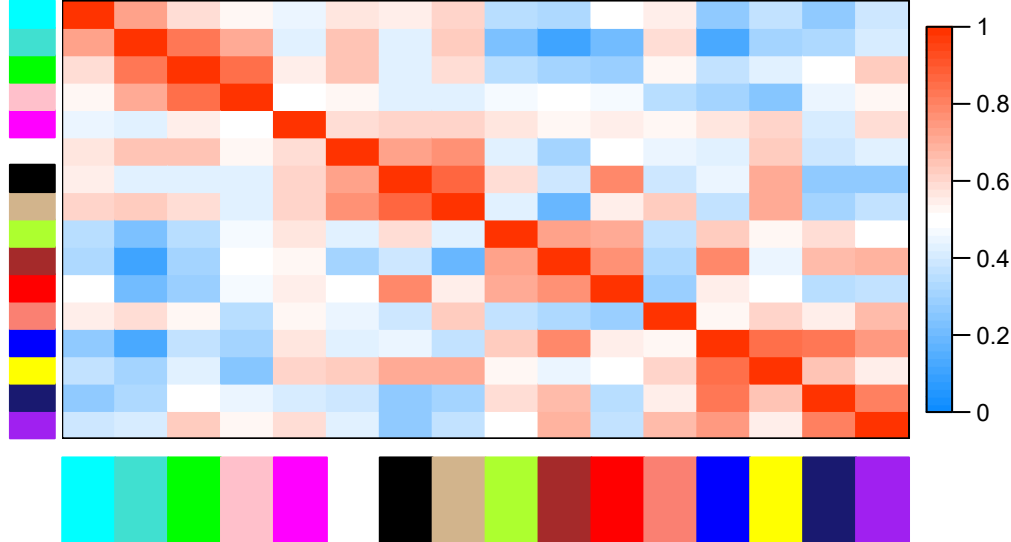

Supplement: Supplementary file 5 — Additional file 5: Figure S5. The module eigengene adjacency heatmap (lower), which indicated the relationship between distinct co-expression modules. The results of module clustering were shown in the upper part. [file 12967_2021_2913_MOESM5_ESM.pdf]

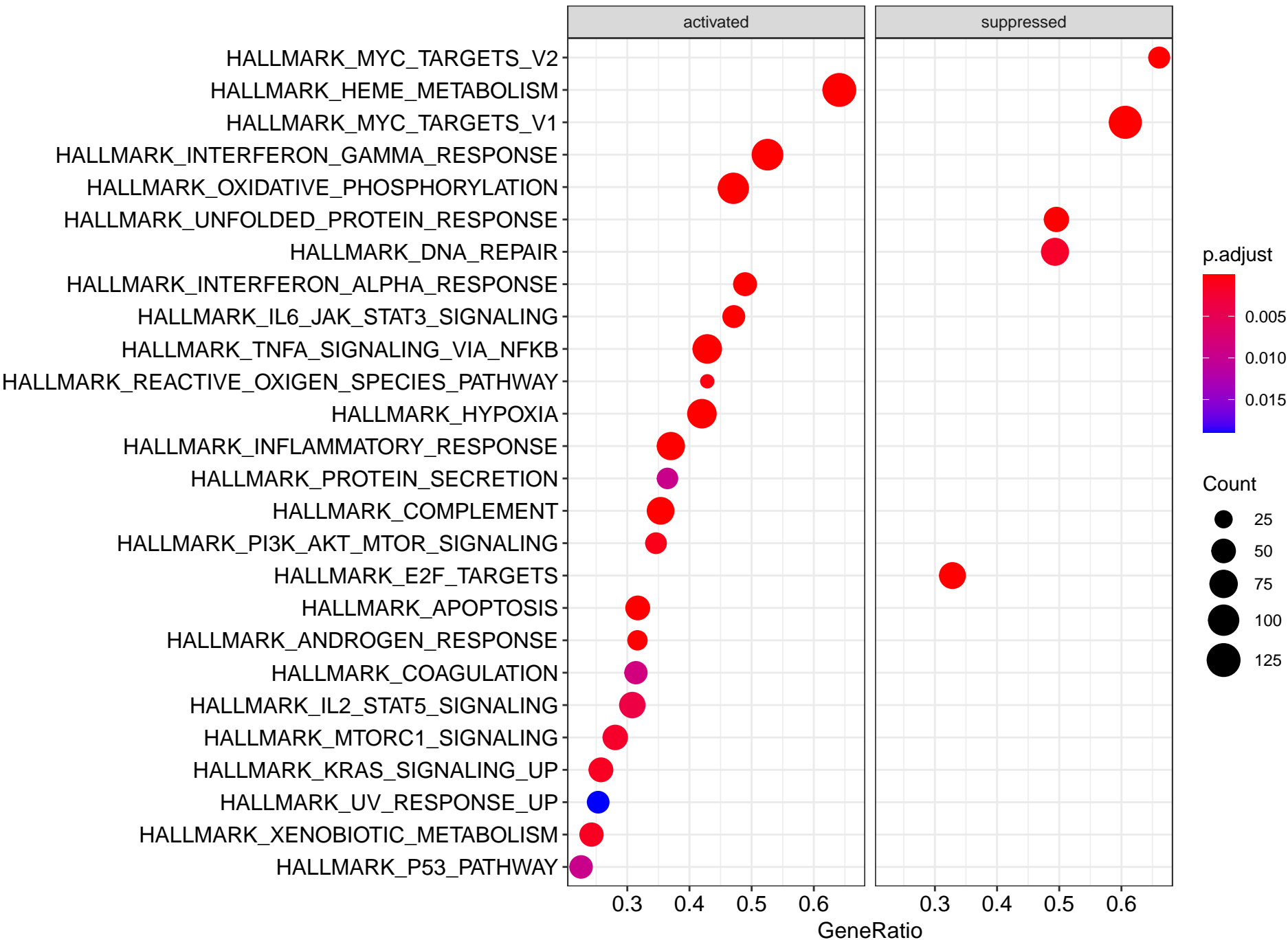

Supplement: Supplementary file 6 — Additional file 6: Figure S6. The dotplot of GSEA results associating with MAPK14 expression. The size of dots represented the count of genes involved in the corresponding pathways. While the color of dots correlated with the -log10(adjusted p value). [file 12967_2021_2913_MOESM6_ESM.pdf]

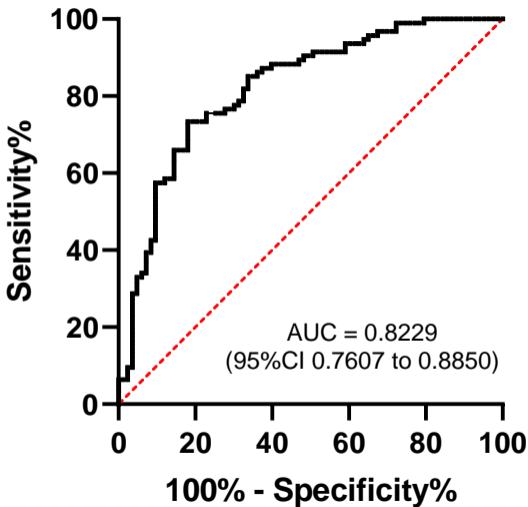

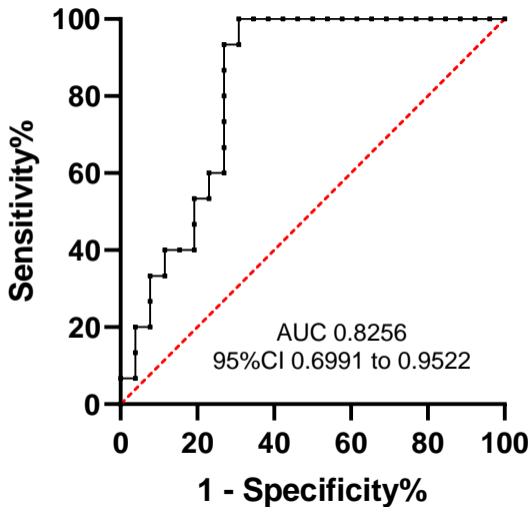

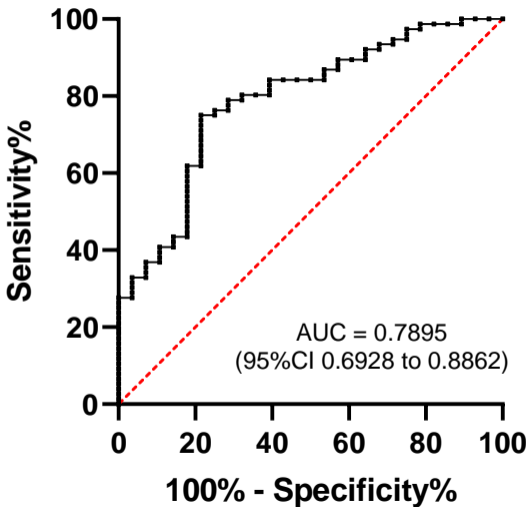

Supplement: Supplementary file 7 — Additional file 7: Figure S7. ROC curve of MAPK14 expression on diagnosis of PV based on the fused GSE61629/GSE26049/GSE57793 dataset (A), GSE54644 (B), and GSE103237 (C). [file 12967_2021_2913_MOESM7_ESM.pdf]
